# Vestibular schwannoma manifesting with hemifacial spasm in a young woman: clinical considerations and tumor removal with hearing preservation. 2-Dimensional operative video

**DOI:** 10.3171/2021.7.FOCVID2099

**Published:** 2021-10-01

**Authors:** Carlos Candanedo, Marrigje A. de Jong, Avner Michaeli, Samuel Moscovici, José E. Cohen, Sergey Spektor

**Affiliations:** Departments of 1 Neurosurgery and; 2 Otolaryngology–Head and Neck Surgery, Hadassah-Hebrew University Medical Center, Jerusalem; and; 3 Surgical Monitoring Services Ltd., Beit Shemesh, Israel

**Keywords:** endoscopic surgery, facial nerve, hearing preservation, hemifacial spasm, vestibular schwannoma, video

## Abstract

Hemifacial spasm (HFS) is a rare presentation of vestibular schwannoma. The authors present their experience with a 27-year-old woman who presented with normal hearing and HFS, which was the single neurological manifestation of an 18-mm vestibular schwannoma. In this challenging situation, the treatment goals were maximal tumor removal with preservation of hearing and facial nerve function and cure of the HFS. The authors achieved these goals, performing complete tumor removal via a retrosigmoid approach, assisted with neurophysiological monitoring and a 45°-angle QEVO endoscope. In the video, they explain the clinical, radiological, and surgical considerations and demonstrate the surgical technique.

The video can be found here: https://stream.cadmore.media/r10.3171/2021.7.FOCVID2099

## Transcript

This is Dr. Carlos Candanedo from the Hadassah-Hebrew University Medical Center in Jerusalem, Israel. This video depicts imaging findings and the resection of a vestibular schwannoma manifesting with hemifacial spasm (HFS) in a young woman, with discussion of clinical considerations and tumor removal with hearing preservation.^1–3^

### 0:39 Patient Clinical History.

A 27-year-old woman, otherwise healthy, presented with a 2-month history of left-side HFS.

### 0:46 Neurological Examination.

On physical examination, besides the intermittent sustained contractions of the perioral facial muscles, there was no other cranial nerve or focal motor deficit.

Preoperative pure tone audiometry showed normal hearing bilaterally, with a speech reception threshold of 10–15 decibels. Preoperative ABR showed a delay in wave III on the left side compared to the right side, indicating affection of the cochlear nerve by the tumor, but hearing was still intact.

### 1:16 Imaging Studies.

Brain MRI revealed a left vestibular schwannoma with an extrameatal tumor diameter of 18 mm compressing the brainstem. Bone window head CT revealed pneumatization of the posterolateral wall of the internal auditory canal (IAC), predicting high risk of postoperative CSF leak.

We decided that the most appropriate treatment would be surgical removal because of the HFS, but given that the patient’s hearing was normal, it was also important to evaluate the chances of hearing preservation. The important prognostic signs in this case are:

- The intracanalicular tumor that did not reach the end of the IAC, as clearly seen on FIESTA images.
- The anatomical position of the labyrinth was convenient, providing the possibility of sufficient IAC drilling to expose the entire tumor without violating the posterior semicircular canal or vestibule, which is also possible to see on MRI FIESTA and CT bone window images.

### 2:14 Patient Positioning.

The surgery was performed with the patient in a right three-quarters prone position, using ABR and 4-channel facial nerve neurophysiology monitoring. We prefer this three-quarters prone position instead of the park-bench position. The head is kept parallel to the floor, while the upper shoulder falls forward with the gravity and facilitates unlimited room for the surgeon’s hands. We also looked for the lateral spread responses, but we found no evidence of this response in our patient. The left hip was also prepped for fat graft harvesting, since we were aware of the possibility of postoperative paradoxical CSF leak.

### 2:51 Approach.

We made a retroauricular curvilinear skin incision and performed a retrosigmoid craniectomy with exposure of the transverse-sigmoid junction. Adequate sealing of the opened mastoid air cells was done with a mixture of bone wax and bone dust. Nowadays, we prefer to build a mold of polymethylmethacrylate (PMMA) bone flap for cranioplasty in situ before opening the dura to avoid the overheating of the cerebellum by flushing off the mold. By doing this, we prevent the toxic monomer that is liberated from being submerged into the intradural spaces, since it is potentially neurotoxic to the cerebellum.^4,5^

### 3:26 Dura Opening.

Since the posterior fossa dura and cerebellum are tensed in this position, we made a small triangular dural incision in the inferior part of the craniectomy and invert the dural flap basally. The otherwise intact dura keeps the cerebellum in place and prevents it from prolapsing. Through this tiny opening, we carefully insert a narrow retractor beneath the cerebellum, elevating it 3–5 mm, enough to open the arachnoid at the edge of the cisterna magna, achieving CSF outflow with cerebellar relaxation. Then, we open dura and you can see the cerebellum is relaxed. To prevent cerebellar trauma, it should not be retracted but elevated. We start from the lower point of the cranial opening, where we have opened the cistern and released CSF. Small bridging veins are identified, coagulated, and transected. We identify the spinal accessory nerve and continue arachnoid dissection around it, and then laterally in the direction of the vagus and glossopharyngeal nerve.

### 4:24 Vestibular Schwannoma Exposure.

Finally, a tumor with a typical appearance of vestibular schwannoma comes into view. Using the Prass probe facial monitor, we check the posterior surface of the tumor to be sure there is no posterior facial nerve location. The dura above the IAC is coagulated and excised. We prefer to open the IAC in the early steps of surgery for the following reasons:

- We obtain more room for surgical manipulation.
- It improves our anatomical understanding.
- Drilling before arachnoid dissections around the tumor prevents the bone dust from being dispersed around the CPA and parabrainstem cisterns since this dust may be a reason for persistent postoperative headaches.

The depth of drilling is measured on the preoperative head CT. We use coarse and regular diamond drills with copious irrigation to prevent thermal damage to the nerves. Mastoid air cells were opened while drilling and obliterated with pieces of bone wax. The dural sleeve inside the IAC is opened and the thin bony shells in the sides of the IAC may be removed with ultrasonic aspiration. Now we debulk the intracranial and intracanalicular part of the tumor with a 1.14-mm precision tip ultrasonic aspiration. After significant tumor debulking, we may proceed to identify the cranial nerves and to dissect the tumor, which we flip away from the brainstem, and start to see the preserved layer of the nerves. Note that the cochlear nerve vestibular nerve and the facial nerve are lying in the same bundle and same direction. The tumor did not split the nerves apart. This is a very important condition for the hearing preservation. From this point, we perform bimanual peeling, not suction and forceps, but with two microforceps, one in each hand of the surgeon. This provided tumor traction with one hand and delicate peeling of the arachnoid or nervous layer with the other hand. Of course, this demands high magnification.

### 6:43 Microvascular Compression Exposure.

Only now we start to see the AICA loop which caused compression of the facial nerve, which was found in its most typical anatomical location, in the inferoventral corner between the tumor and the brainstem. We separate the AICA loop from the facial nerve. There was obvious compression, which explained the hemifacial spasm, the patient’s main complaint. The tumor had compressed the facial nerve against the AICA. The entire nervous layer containing facial and cochlear nerves was very well preserved and covered with microcottonoid. We proceeded with the removal of tumor remaining in the IAC. It is important to mention that dissection should be done in the peripheral direction from the brainstem to the ending of the IAC and not the opposite, since traction away from the fundus may cause trauma and rupture of the fine nervous fibers where they penetrate into cochlea and cause a hearing loss. Wide IAC opening enabled bimanual dissection even inside the canal.

### 8:03 Microinspection Tool Insertion.

We removed the last visible tumor piece with an angled curette and inserted a QEVO endoscopic microinspection tool of a 45° angle.^6^ This revealed obvious residual tumor in the fundus of the IAC. We drilled additional 3 mm of the lateral IAC and removed this tumor remnant. Repeat examination through QEVO now confirmed complete tumor removal. The facial and cochlear nerve are well preserved. The facial nerve responded to stimulation with 0.02 mA from the exit area. Since the patient had HFS and there was an obvious compression by the AICA, we placed a tiny Teflon polymer pledget between the artery and the nerve. A piece of fat harvested from the patient’s hip was inserted to pack the drilled area of the IAC, without compressing the nerves, and a mixture of cryoprecipitate and thrombin was added to help sealing the opened air cells.

### 9:15 Closure.

Watertight dural closure was complete with Lyoplant, which is a dura matter substitution from lyophilized bovine collagen and Prolene 5-0. This is a fusion image of the preoperative MRI, and postoperative CT shows in red the drilled area next to the IAC, in yellow the part of the tumor removed microscopically, and in light blue the part removed with the endoscope.

### 9:41 Postoperative Course.

The pathology report was consistent with schwannoma, as expected. Following surgery, the patient’s HFS resolved and she had no evidence of facial palsy or subjective hearing loss. Postoperative pure tone audiometry revealed a new-onset unilateral high-tone perceptive hearing loss at 6–8 kH of 100–105 dB on the left side, but speech perception thresholds were unaffected. Postoperative brain MRI showed the fat in the surgical area sealing the opened mastoid air cells around the IAC and complete removal of the tumor, including the intracanalicular segment.

## Acknowledgments

We thank Shifra Fraifeld, a senior medical writer and research associate, for her editorial assistance with the preparation of this transcript.

## Disclosures

The authors report no conflict of interest concerning the materials or methods used in this study or the findings specified in this publication.

## Author Contributions

Primary surgeon: Spektor. Assistant surgeon: Candanedo. Editing and drafting the video and abstract: Spektor, Candanedo. Critically revising the work: all authors. Reviewed submitted version of the work: Spektor, Candanedo, Michaeli, Moscovici, Cohen. Approved the final version of the work on behalf of all authors: Spektor. Supervision: Spektor, Candanedo, Moscovici, Cohen. Otolaryngology consultation: de Jong. Neurophysiology monitoring: Michaeli.
